# Knowledge, attitudes and demographic drivers for COVID-19 vaccine hesitancy in Malawi

**DOI:** 10.1038/s41598-024-60042-5

**Published:** 2024-04-26

**Authors:** Yamikani Ndasauka, Halima Sumayya Twabi, Jimmy Kainja, Anthony Mavuto Gunde, Catherine Makhumula-Mtimuni

**Affiliations:** 1 Department of Philosophy, University of Malawi, Zomba, Malawi; 2 Department of Mathematical Sciences, University of Malawi, Zomba, Malawi; 3 Language and Communication Skills Department, University of Malawi, Zomba, Malawi; 4 Department of Fine and Performing Arts, University of Malawi, Zomba, Malawi

**Keywords:** Health policy, Public health

## Abstract

This study assessed the association among knowledge, attitudes and uptake of COVID-19 vaccine in Malawi, examining demographic factors influencing these variables. The study employed a quantitative research design. It thus sampled 394 participants from Malawi’s three districts of Zomba, Dowa and Nkhatabay. Results showed that 163 (41.4%) participants had low levels of knowledge of COVID-19 vaccine, 231 (58.6%) had high levels of knowledge, 237 (60.2%) had a positive attitude, and 156 (39.8%) had a negative attitude towards the COVID-19 vaccine. On vaccine uptake, the study found that only 29 (17%) male and 52 (23%) female participants had received the vaccine. Further, participants with low levels of knowledge and a positive attitude towards the vaccine were 5.9 times more likely (*p*-value = 0.001) to be vaccinated than those with low levels of knowledge and negative attitudes towards the vaccine. On the other hand, those with high knowledge and a positive attitude towards the vaccine were 8.2 times more likely (*p*-value < 0.001) to be vaccinated compared to those with low knowledge and negative attitudes towards the vaccine. The findings highlight the importance of vaccine-related knowledge and attitudes in shaping uptake and reveal disparities across demographic groups. To improve vaccination coverage in Malawi, targeted interventions focusing on enhancing COVID-19 vaccine knowledge, addressing attitudinal barriers, and countering misinformation are needed. Strategies should be tailored to reach populations with lower uptake, such as males, younger adults, and those with lower education levels. Strengthening public health messaging, engaging community leaders, and building trust in healthcare systems are crucial for promoting widespread acceptance and uptake of COVID-19 vaccines in Malawi.

## Introduction

The COVID-19 pandemic has been an unprecedented global health crisis, resulting in over 6 million confirmed deaths worldwide as of July 2023^1^. Mitigating the impact of the pandemic has required multifaceted public health strategies, including non-pharmaceutical interventions like lockdowns and social distancing and pharmaceutical interventions like diagnostic tests, treatments, and vaccines. Developing and deploying safe and effective vaccines have been crucial for controlling viral transmission and reducing severe disease and mortality^2^. However, achieving high and equitable vaccination coverage globally has proven challenging. A significant barrier has been vaccine hesitancy, defined by the World Health Organization (WHO) as the delay in accepting or refusing vaccination despite availability^2^. Vaccine hesitancy is a complex and context-specific phenomenon shaped by complacency, convenience, confidence, risk calculation, and collective responsibility^3^. Complacency arises when individuals underestimate the risks of vaccine-preventable diseases. Convenience refers to barriers like accessibility, affordability, and willingness to invest time and resources to get vaccinated. Confidence involves trust in the effectiveness and safety of vaccines, the health system offering them, and policymakers deciding on their rollout. Risk calculation is the process of individuals assessing risks and benefits associated with vaccination based on available information. Collective responsibility is the commitment to protect others through vaccination, which declines if people perceive diseases as less common or dangerous^3^.

Vaccine hesitancy has challenged immunisation programs for decades and is listed by WHO as one of the top ten threats to global health^2^. Concerns around new vaccines are a historical phenomenon, even for the first vaccine against smallpox in the nineteenth century^4^. Common drivers include religious beliefs, philosophical reasons, safety concerns, and a desire for more information^5^. The rapid development and deployment of COVID-19 vaccines during the pandemic intensified pre-existing vaccine hesitancy trends globally. Surveys found decreased vaccine acceptance associated with concerns around effectiveness, side effects, and the speed of clinical trials and emergency authorisation^6^. Misinformation and conspiracy theories spreading rapidly through social media also bred mistrust in scientific institutions and health authorities^7^.

Africa has a unique precedent of vaccine hesitancy rooted in structural barriers around vaccine delivery, social marginalisation, and erosion of public trust. Historical failures to involve local communities and strengthen health systems have plagued immunisation programs^8^. Inadequate cold chain capacities, lack of routine vaccination schedules, and absence of multisector coordination limited vaccine availability and accessibility across the continent^9^. The effects of structural racism, gender inequities, poverty, and weak health literacy exacerbated hesitancy among marginalised groups. Unmet expectations around past vaccination campaigns and experimental drug trials without consent further diminished trust and demand^10^. This legacy continues to impact vaccine decision-making, necessitating context-specific investigations.

Malawi’s COVID-19 vaccine program reflects broader challenges around hesitancy and equitable coverage in Africa. Malawi faces substantial health and development challenges as a low-income country with 18 million people. Over 70% live below the poverty line, and only half have access to essential health services^11^. Malawi detected its first COVID-19 case in April 2020 and initiated its vaccination campaign in March 2021 after joining the COVAX facility. The goal was to immunise 20% of the population by the end of 2021 and 70% by the end of 2022^12^.

Malawi has undertaken concerted efforts to make COVID-19 vaccines available and accessible to its population despite being a low-income country with significant health system constraints. To expand access, the government implemented mass vaccination campaigns providing free shots at health centres, schools, markets, border posts, places of worship, and mobile clinics countrywide^13^. The health ministry collaborated with professional associations and external partners to communicate and broaden people's access to the vaccine. For instance, it rolled out a "Vaccinate My Village" project, which engaged traditional leaders and community volunteers to mobilise uptake through risk communication and reducing access barriers^14^.

Despite these efforts, as of July 2023, only around 20% of the total population has been fully vaccinated^1^. Low vaccine uptake despite relative availability and free access indicates significant COVID-19 vaccine hesitancy. Reported reasons include misconceptions about safety fuelled by rumours and disinformation^15^. Religious beliefs such as COVID-19 vaccines being the mark of the beast mentioned in the Bible’s Book of Revelation have also bred resistance, especially in rural areas^16^. Safety concerns stem from rapid vaccine development and a lack of trust in Western pharmaceutical companies^37^. Structural barriers like distances to vaccination sites and the absence of national IDs hinder access for rural and marginalised groups^4^.

There has been minimal research on vaccine hesitancy in Africa. In their scoping review of vaccine hesitancy in Africa, Ackah et al.^9^ found only about 100 studies conducted in Africa, most of which were in Ethiopia (32%). However, Africa has a historical precedent of vaccine hesitancy—hesitancy fuelled by a lack of community involvement, adequate vaccine infrastructure, multisectoral collaboration, and routine immunisation schedules acts as part of a robust one-health modality. This study was premised on this understanding and sought to contribute to this literature by examining the prevalence and associations of COVID-19 vaccine knowledge, attitudes, and uptake in Malawi. The findings will provide actionable insights to guide public health messaging and interventions for improving Malawi's COVID-19 vaccine acceptance and coverage. Successful COVID-19 vaccination will reduce disease burden and prevent loss of life in subsequent pandemic waves. More broadly, the lessons can strengthen routine immunisation programs and health service delivery for marginalised populations in Malawi. With emerging variants and widening immunity gaps, addressing vaccine equity remains an ethical imperative for collective health and well-being.

## Methodology

### Study population and design

The study followed a community-based cross-sectional study design. This study was conducted among selected individuals aged 18 years and older within three districts in Malawi: Zomba in the eastern region, Nkhata-bay in the northern region and Dowa in the central region. In Zomba, participants were selected from the following communities: Chinamwali, Sadzi, Mpondabwino, M’bwana and Songani, while in Dowa, the following communities were sampled: Mponela, Madise and Dowa turnoff and in Nkhata-Bay, participants were selected from Chintheche and Tukombo. Convenience sampling was used to select the participants as probabilistic sampling would have been hard to attain due to limitations in obtaining a reliable sampling frame at a district level during the COVID-19 pandemic period. The study utilised a well-structured survey questionnaire (see Data Collection Tools_ESM) to quantitatively capture the demographic details, vaccine knowledge, attitudes toward the vaccine, and vaccine uptake.

### Sample size determination

This sample comprised individuals aged 18 years and above. The sample size was calculated using the finite population sample size formula based on the national population of people 18 years and older, as reported in the 2018 census report, using the following formula:

$$n = \left[ {\left( {\left( {z^{2} \times p\left( {1 - p} \right)} \right)/e^{2} } \right) \times N\left] / \right[\left( {\left( {z^{2} \times p\left( {1 - p} \right)} \right)/e^{2} } \right) + \left( {N - 1} \right)} \right]$$

where z is the level of confidence (95%); e is the acceptable level of error (0.05); $p$ is the fraction of responses that we are interested in and is estimated from previous studies or estimated by 0.5 in this case where such information is not documented; N is the known population size of adults (18 years above) in Malawi- N = 8,700,000. Based on these values, the sample size (n) was initially calculated to be 384. The sample size was increased by 2.5% to a final sample of 394 to account for potential non-responses. This sample was distributed among the three proposed districts using probability proportional to the population size within the district.

### Data collection and assurance of data quality

Data were collected from the 14th to the 21st of December 2021 through face-to-face interviews using a structured questionnaire. The data were collected electronically using tablet applications, specifically ODK/KOBO. Three research team members developed the questionnaire to ensure consistency and clarity of the questions. The questions were formulated after an extensive literature search and review. The questionnaire was translated into the local language and reviewed by a language expert. The supervisor and data collection teams underwent training to understand the questions within the data collection tool and identify any ambiguous questions and responses. The questionnaire was tested through a pilot study among purposively selected individuals. Questions that were unclear, ambiguous and difficult were revised and modified. Supervisors were deployed throughout the data collection to thoroughly supervise the data analysis process and collect complete questionnaires after assessing their accuracy. The collected data was stored in a database, and before processing, it was checked for any double-entry errors in coding for accuracy and consistency.

### Socio-demographic variables

Some socio-demographic questions were asked during the survey, including age (later categorized as young [18–24 years], [25–34 years], [35–44 years] and adult [> 45 years]), sex (Male/Female), marital status (Never married/Married/Divorced/Separated/Widowed), level of education (No education/Primary, Secondary/Post-secondary), Occupation (Unemployed/Employed/Self-employed) and religion (Christians/Muslims, Other).

### Operational definitions and measurements

The knowledge on vaccines comprised four questions, with one having two possible responses (Yes and No). Examples include, “Have you heard of the COVID-19 vaccine?” A ‘yes’ response was coded as 1, while the 'no' responses were coded as 0. Two other questions had five possible responses (Strongly disagree, Disagree, Neutral, Agree, and Strongly Agree). An example is, “Even though there is a vaccine, other preventive measures are important” (see details in Table 1). A response that strongly agrees or agrees that the vaccine is safe or that other preventive measures are needed apart from the vaccine was coded as 1, and a wrong answer was coded as 0. Not answering or not knowing was classified under neutral and calculated as 0. The total score for knowledge of vaccines was obtained by summating the raw scores of the four items, which ranged from 0 to 4, with the higher score indicating an adequate understanding of COVID-19 vaccinations. Overall, knowledge of the COVID-19 vaccine was categorised as low if the score was ≤ 50% and high if the score was above 50%.

**Table 1:** Knowledge of the COVID-19 vaccine.

	Frequency	Percent
Have you ever heard of the COVID-19 vaccine?		
Yes	363	92.1
No	31	7.9
What does the COVID-19 vaccine prevent? *		
It prevents one from catching COVID-19	156	43
It prevents one from getting severely sick from COVID-19	167	46
It does not prevent one from anything	19	5.2
Other specify	3	0.8
Do not know	18	5
Even though there is a vaccine, other preventive measures are important. *		
Strongly agree	154	42.4
Agree	179	49.3
Neutral	12	3.3
Disagree	8	2.2
Strongly disagree	9	2.5
Other specify	1	0.3
The COVID-19 vaccine is safe		
Strongly agree	57	14.5
Agree	119	30.2
Neutral	68	17.3
Disagree	109	27.7
Strongly disagree	41	10.4

*Total missing = 31.

The attitude towards the vaccines comprised 6 items with five possible responses (Coded from 1 to 5. Strongly Disagree, Disagree, Neutral, Agree, and Strongly Agree). An example of a question was- COVID-19 vaccine is essential to me? (see details in Table 2). Negative questions were reverse-scored. The cumulative score for all six questions ranged from 6 to 30 (for positive responses). Hence, a total score ≤ 15 was considered a negative attitude, and a score > 15 was considered a positive attitude.

**Table 2:** Attitudes towards the COVID-19 vaccine.

Attitudes	Strongly agree	agree	Neutral	Disagree	Strongly disagree
	N (%)	N (%)	N (%)	N (%)	N (%)
The COVID-19 vaccine is important to me	95 (24%)	170 (43%)	41 (10%)	72 (18%)	16 (4%)
The COVID-19 vaccine is important for my community	106 (27%)	182 (46%)	47 (12%)	49 (12%)	10 (3%)
Every Malawian must be vaccinated	96 (24%)	153 (39%)	60 (15%)	66 (17%)	19 (5%)
I would recommend that my family and friends get the COVID-19 vaccine	115 (29%)	169 (43%)	29 (7.4%)	59 (15%)	22 (6%)
I would prefer to acquire natural immunity against COVID-19	80 (20%)	110 (28%)	61 (16%)	101 (26%)	42 (11%)
The COVID-19 vaccine will affect one’s health in the long run	67 (17%)	73 (19%)	76 (19%)	120 (31%)	58 (15%)

### Data management and analysis

Data were cleaned and analysed using STATA version 15 software. We calculated the prevalence of participants’ awareness and attitudes towards COVID-19 vaccine and COVID-19 vaccination uptake and explored how these were distributed across demographic factors, e.g., gender and age. Inferential statistics were also employed to develop models explaining the role of various factors on COVID-19 knowledge and attitudes towards the vaccine. Bivariate analysis was done using a Chi-square statistic to identify demographic factors associated with knowledge, attitude, and vaccine uptake. A multivariable logistic regression model was fit to explain factors (cultural and demographic) influencing vaccine hesitancy behaviours. In addition, factors deemed as purported confounders were controlled by including them in the logistic regression model. We conducted a multivariable analysis using a logistic regression model to determine demographic factors influencing the knowledge of COVID-19 and attitudes towards the vaccine. We included variables that were significant in the bivariate analysis. However, we also added variables that we deemed essential factors based on literature (see^17–19^).

### Ethical considerations

The study fully adhered to ethical standards expressed in the Declaration of Helsinki. Before the commencement of the study, relevant approval was given by the University of Malawi Research Ethics Committee (UNIMAREC) Protocol No: P.11/21/101, district commissioners and appropriate community leaders. Participation in the survey was voluntary. The study obtained written informed consent from participants 18 years old and above. Participants who could not sign their consent forms due to other challenges were asked to use their fingerprints to give consent. Responses from participants were kept strictly confidential. No physical, discomfort or psychological risks were anticipated from participating in this study. Nevertheless, participants were notified that they could skip it or withdraw from the study if they felt uncomfortable with a question.

## Results

### Demographic characteristics

A total number of 394 participants consented was interviewed (100% response rate) and included in the analysis. Table 3 shows the demographic characteristics of the study participants. Of the 394, 43% were males, and 57% were females; 52% were from Zomba, 24% were from Dowa, and 23% were from Nkhatabay. Most participants had secondary education (49%), and a majority were Christians (91%). A highest proportion of the participants were married (59.9%) and and a majority were unemployed (46.5%). The mean age was 31 years, with a standard deviation of 13. The minimum age was 18, and the maximum age was 80.

**Table 3:** Demographics of Study Participants.

Demographic characteristics	Freq	Percent (%)
District		
Zomba	206	52
Dowa	97	25
Nkhatabay	91	23
Age category		
18–24 years	151	38
25–34 years	127	32
35–44 years	58	15
45 years above	58	15
Gender		
Males	168	43
Females	226	57
Education category		
None	11	3
Primary	157	40
Secondary	193	49
Post-secondary	33	8
Religion		
Christian	359	91.1
Muslim/other	35	8.9
Marital status		
Never married	114	28.9
Married	236	59.9
Widow	16	4.1
Separated/divorced	28	7.1
Occupation		
Unemployed	183	46.5
Employed	61	15.5
Self-employed	150	38

### Knowledge of the COVID-19 vaccine

Out of the 394 participants, 163 (41.4%) had low knowledge of the COVID-19 vaccine, 231 (58.6%) had high knowledge, and 176 (45%) agreed that the vaccine was safe, while 150 (38%) disagreed that the vaccine was safe. Of the 394 participants, 363 (92.1%) had heard of the COVID-19 vaccine. Among the participants, 156 (43%) correctly stated that the vaccine prevents one from getting severely sick. In comparison, 156 (43%) incorrectly said that it prevents one from catching COVID-19, and 5.2% stated that it did not prevent one from anything. 154 (42.4%) and 179 (49.3%) strongly agreed and agreed that even though there is a vaccine, other preventative measures are important. Of the respondents, 57 (14.5%) strongly agreed, and 119 (30.2%) agreed that the vaccine was safe.

### Attitudes towards COVID-19 Vaccine

Out of the 394 respondents, 237 (60.2%) had a positive attitude towards the COVID-19 vaccine, while 156 (39.8%) had a negative attitude. According to Table 2, 274 (72%) participants agreed they would recommend their families get vaccinated, while 249 (63%) believed every Malawian must be vaccinated. 190 (48%) would prefer to acquire natural immunity against COVID-19, whilst 139 (36%) thought that the vaccine would affect their health in the long run.

### Demographic factors and knowledge and attitudes towards the vaccine

Table 4 presents the Chi-square test results to determine the significant relationship between knowledge and attitudes towards the COVID-19 vaccine and demographic variables.

**Table 4:** Association among knowledge of the COVID-19 vaccine, attitude towards the vaccine and Demographic Factors.

	Knowledge			Attitude		
	Low	High	p-value	Negative	Positive	p-value
Total (n = 394)	181	213		157	237	
Gender						
Male	69 (41%)	99 (58.9%)		58 (34.5%)	110 (65.5%)	
Female	112 (50%)	114(50.4%)	0.095	99(43.8%)	127(56.2%)	0.063
District						
Zomba	85 (41%)	121 (59%)		89(43%)	117 (57%)	
Dowa	47 (49%)	50 (51%)		24 (25%)	73 (75%)	
Nkhatabay	49 (54%)	42 (46%)	0.113	44 (48%)	47 (52%)	0.002*
Age						
18–24 years	78 (40%)	73 (60%)		61 (40%)	90 (60%)	
25–34 years	56 (43%)	71 (57%)		54 (43%)	73 (57%)	
35–44 years	22 (36%)	36 (64%)		21 (36%)	37 (64%)	
45 years above	25 (36%)	33 (64%)	0.279	21 (36%)	37 (64%)	0.792
Education level						
None	9 (81%)	2 (18%)		6 (55%)	5 (45%)	
Primary	92 (59%)	65 (41%)		72 (46%)	85 (54%)	
Secondary	76 (39%)	117(61%)		70 (36%)	123 (64%)	
Post-secondary	4 (12%)	29 (88%)	< 0.001*	9(27%)	24(73%)	0.087
Religion						
Muslim/Other	18 (51%)	17 (49%)		11 (31%)	24 (69%)	
Christian	163 (45%)	196 (55%)	0.495	146 (41%)	213 (59%)	0.287
Marital status						
Never married	47 (41%)	67 (59%)		44 (39%)	70 (61%)	
Married	117 (49%)	119 (51%)		96 (41%)	140 (59%)	
Widowed	6 (38%)	10 (62%)		6 (38%)	10 (62%)	
Separated/divorced	11 (39%)	17 (61%)	0.357	11 (40%)	17 (60%)	0.980
Occupation						
Unemployed	89 (49%)	94 (51)		80 (44%)	103 (56%)	
Employed	20 (33%)	41 (67)		23 (38%)	38 (62%)	
Self-employed	72 (48%)	78 (52)	0.080	54 (36%)	96 (64%)	0.335

*Significant values.

According to Table 4, education was significantly associated with knowledge of the COVID-19 vaccine (p-value < 0.05. However, no significant difference in knowledge of the COVID-19 vaccine was observed across the districts, gender, and age categories. We then conducted a multivariable analysis using a logistic regression model to determine demographic factors influencing the knowledge of the COVID-19 vaccine and attitudes towards the vaccine. Results are presented in Table 5.

**Table 5:** Multivariable Analysis- demographic factors associated with knowledge and attitudes towards the COVID-19 vaccine.

	Knowledge of COVID-19 vaccine			Attitude towards COVID-19 vaccine		
	Adjusted Odds ratio	95% Confidence interval	*p*-value	Adjusted odds ratio	95% Confidence Interval	*p*-value
Gender						
Male	1.00			1.00		
Female	0.84	(0.52–1.35)	0.478	0.75	(0.46–1.20)	0.225
District						
Zomba	1.00			1.00		
Dowa	0.80	(0.47–1.33)	0.382	2.71	(1.54–4.73)	< 0.001*
Nkhatabay	0.67	(0.38–1.16)	0.153	0.80	(0.45–1.33)	0.343
Age category						
18–24 years	1.00			1.00		
25–34 years	1.63	(0.91–2.94)	0.098	0.86	(0.48–1.51)	0.594
35–44 years	2.54	(1.19–5.38)	0.015*	1.15	(0.55–2.41)	0.710
45 above	2.32	(1.07–5.07)	0.034*	1.41	(0.65–3.08)	0.380
Education level						
No education/primary	1.00			1.00		
Secondary/post-Secondary	2.75	(1.78–4.26)	< 0.001*	1.72	(1.11–2.67)	0.016*
Religion						
Muslim/other	1.00			1.00		
Christian	1.30	(0.61–2.78)	0.488	0.65	(0.30–1.44)	0.290
Marital Status						
Never married	1.00			1.00		
Married	0.65	(0.35–1.19)	0.163	1.10	(0.60–2.00)	0.780
Widowed/separated/divorced	0.95	(0.38–2.39)	0.910	1.13	(0.46–2.80)	0.784
Occupation						
Unemployed	1.00			1.00		
Employed	1.49	(0.76–2.92)	0.244	1.14	(0.59–2.19)	0.692
Self-employed	0.84	(0.51–1.39)	0.500	1.45	(0.88–2.40)	0.148

*Significant values.

According to Table 5, education and age were associated with knowledge of COVID-19 vaccines. Individuals with a secondary or post-secondary education (OR: 2.75; 95% CI [1.78–4.26]) were more likely to have adequate knowledge of COVID-19 compared to those who had no education or primary education. In addition, individuals who were 35–44 years years (OR: 2.54; 95% CI [1.19–5.38]) and 45 years above (OR: 2.32; 95% CI [1.07–5.07]) were more likely to have adequate knowledge of COVID-19 compared to those aged 18–24 years old.

Further, results showed an association between having an attitude towards the vaccine and residing in a particular district. However, the association was only significant for the Dowa district. Individuals from Dowa were more likely to have a positive attitude towards the COVID-19 vaccines than those in Zomba (OR: 2.71; 95% CI [1.54–4.73]). However, no association with a positive attitude towards the vaccine was observed for age, education and gender.

### COVID-19 vaccination uptake

Level of vaccination uptake was also assessed by gender. A total of 81 participants had received at least one dose of the COVID-19 vaccine. Out of the 81, 29 (17%) were male and 52 (23%) were female. Of the 81 participants who received the vaccine, only 47 had received a full dose, representing 12% of the total sample. However, there was no significant difference in vaccination uptake between males and females.

Table 6 presents factors associated with vaccination uptake. As shown in Table 6, females were more likely to get vaccinated than males (Odds ratio = 1.99, *p*-value = 0.024). Further, participants with secondary or post-secondary education were more likely to get vaccinated than those with no education or primary education (Odds ratio = 1.8, *p*-value = 0.041). Participants who were employed were more likely to be vaccinated than those not employed (Odds ratio = 2.56, *p*-value = 0.016).

**Table 6:** Demographic Factors Associated with Vaccination Uptake.

	Adjusted Odds ratio	95% Confidence Interval	*p*-value
Gender			
Male	1.00		
Female	1.99	(1.10–3.58)	0.024*
District			
Zomba	1.00		
Dowa	1.02	(0.54–1.92)	0.953
Nkhatabay	0.66	(0.33–1.33)	0.245
Age			
18–24 years	1.00		
25–34 years	0.75	(0.35–1.60)	0.458
Above 35 years	2.14	(0.99–4.63)	0.054
Education			
None/primary	1.00		
Secondary/post-secondary	1.81	(1.02–3.14)	0.041*
Religion			
Muslim/other	1.00		
Christian	1.44	(0.50–4.12)	0.499
Marital status			
Never married	1.00		
Married	1.15	(0.52–2.53)	0.730
Widowed/separated/divorced	1.39	(0.46–4.18)	0.561
Occupation			
Unemployed	1.00		
Employed	2.56	(1.19–5.50)	0.016*
Self-employed	1.54	(0.82–2.90)	0.181

*Significant values.

### The role of knowledge and attitudes on vaccination uptake

We assessed the associations between knowledge of COVID-19 and attitudes towards the vaccine on vaccination uptake. As shown in Table 7, we observed a significant association between knowledge of COVID-19, attitude towards the vaccine and uptake of vaccination. Individuals with high knowledge were 1.47 times more likely to get vaccinated than those with low knowledge (p-value < 0.001). Similarly, those with a positive attitude towards the COVID-19 vaccine were more likely to get vaccinated than those with a negative attitude (OR: 1.97; 95% CI [1.24–2.70]).

**Table 7:** Association between vaccination knowledge and attitudes and vaccination uptake.

	Adjusted Odds ratio	95% Confidence Interval	p-value
Knowledge of COVID-19			
Low	1.00		
High	1.40	(0.37–5.29)	0.627
Attitude towards vaccine			
Negative	1.00		
Positive	1.97	(1.24–2.70)	< 0.001*

*Significant values.

We examined the relationship between COVID-19 vaccination knowledge and attitude on uptake, stratified by gender, education, and age. Females with high knowledge of the vaccine were more likely to get vaccinated. In contrast, individuals with only a primary education and high knowledge were less likely to get vaccinated. Both males and females, those with a primary or secondary/post-secondary education and individuals across all ages with a positive attitude towards the vaccine were more likely to be vaccinated. (see Table 8).

**Table 8:** Association between knowledge and attitudes towards vaccine and vaccine uptake segregated by gender, education and age.

	Knowledge	Attitude
	High	Positive
	Unadjusted OR (95% CI) (p-value)	Unadjusted OR (95% CI) (p-value)
Gender		
Male	1.40 (0.61–3.23) (0.429)	5.67(1.64–19.7) (0.006)
Female	1.99 (1.10–3.78) (0.034)	8.80(3.57–21.7) (< 0.001)
Education		
Primary	0.32 (0.11–0.91) (0.033)	5.94(1.94–18.2) (0.002)
Secondary/post-secondary	1.21 (0.63–2.33) (0.563)	9.37 (3.24–27.1) (< 0.001)
Age in categories		
18–24 years	1.32 (0.55–3.17) (0.534)	5.88 (1.67–20.7) (0.006)
25–34 years	1.22 (0.46–3.23) (0.688)	5.16 (1.43–18.6) (0.012)
Above 35 years	2.38 (1.10–5.56) (0.448)	11.1 (3.13–38.9) (< 0.001)

In addition, we assessed the relationship between knowledge of the vaccine and attitudes towards the vaccine, as they may be related. Results showed that the odds of a positive attitude towards a vaccine were high (1.8 times more likely, 95% CI: [1.2, 2.7]) among those with high knowledge of the vaccine compared to those with low knowledge (See Table 9).

**Table 9:** Association between vaccination attitude and knowledge.

	Odds ratio	95% Confidence Interval	p-value
Knowledge of COVID-19 vaccine			
Low	1.00		
High	1.81	(1.20 – 2.72)	< 0.001*

*Significant values.

As shown in Tables 7 and 9, vaccination knowledge is positively associated with attitude towards the vaccine and uptake of vaccination; hence, it may act as a moderating factor for the relationship between attitude towards the vaccine and vaccination uptake. Thus, we further assessed the moderating effect of attitudes towards the pandemic on the association between attitudes towards the vaccine and vaccination uptake, controlling for confounders of age, gender and education level (Table 10). Table 10 estimates how much the effect of attitude towards a vaccine on vaccination uptake varies between individuals with vaccination knowledge (versus) those without knowledge while adjusting for the socio-demographics in the study. Participants with low knowledge of the vaccine and a positive attitude towards the vaccine were 5.9 times more likely (*p*-value = 0.001) to be vaccinated than those with low knowledge of but negative attitudes towards the vaccine. On the other hand, those with high knowledge of the vaccine and a positive attitude towards the vaccine are 8.2 times more likely (*p*-value < 0.001) to be vaccinated compared to those with low knowledge of and negative attitudes towards the vaccine. The results suggest that knowledge of the COVID-19 vaccine significantly influences one's propensity to adopt a positive attitude towards COVID-19 vaccination.

**Table 10:** The moderating effect of knowledge of the COVID-19 vaccine.

Interaction	Adjusted odds ratio	*p*-value	95% CI
Knowledge × Attitude vaccine			
Low × Negative	1.00		
Low × Positive	5.87	0.001	2.08 – 16.54
High× Negative	0.74	0.686	0.17 – 3.24
High × Positive	8.20	< 0.001	3.04 – 22.05

## Discussion

The study assessed knowledge of COVID-19 and attitudes towards the COVID-19 vaccines and their association with vaccine uptake in Malawi. The results show that most participants had high level knowledge of the pandemic and a positive attitude towards the COVID-19 vaccines. Gender and education were significantly associated with attitudes towards the vaccine in that females were less likely to have a positive attitude towards the vaccine than males. Further, despite the availability of the COVID-19 Vaccine, only a small population had received the full dose of the vaccine, with more females getting vaccinated than males. Older participants were more likely to get vaccinated than younger participants. There was a significant association between knowledge, attitude, and vaccination uptake. Individuals with high knowledge of the pandemic and a positive attitude towards the vaccine were more likely to get vaccinated than those with a negative attitude. However, there was a non-significant likelihood that individuals with high knowledge of the pandemic but negative attitudes towards the vaccine would get vaccinated. This means that knowledge alone plays a significant but minor role in influencing the decision to vaccinate (see^36^).

Our findings that knowledge and positive attitudes towards the COVID-19 vaccine were relatively higher than negative attitudes are consistent with several previous studies in other contexts. For example, a study in Bangladesh by Islam et al.^20^ found heightened awareness and moderately positive attitudes, with only 22% expressing unwillingness to vaccinate. Similarly, a multi-country study across Africa and the Middle East by El-Far et al.^21^ reported general positive sentiment despite some concerns. Studies from Ethiopia^22^ and South Africa^23^ also found favourable disposition and acceptance superseded vaccine hesitancy.

The moderately high levels of COVID-19 vaccine knowledge and positivity in our Malawian sample suggest people recognised the importance of immunisation for controlling the pandemic. Widespread public health messaging and awareness campaigns likely increased vaccine literacy and demand. Malawi's government and other organisations utilised diverse communication channels to disseminate information about COVID-19 and vaccines through television, radio, print media, social media, and community mobilisation^12^. Also, the long history of childhood immunisation programs in Malawi, with roots in the 1980s, has cultivated broader societal acceptance of the value of vaccines for preventing infectious diseases^24^.

Our finding that females expressed more negative attitudes towards COVID-19 vaccines than males aligns with several previous studies. Surveys across multiple countries, including the US, UK, Italy, Bangladesh, and the UAE, found women were more likely to be hesitant or unwilling to get vaccinated compared to men^20,25–30^. Common concerns included fears about vaccine safety, side effects, fertility impacts, and overall risk–benefit calculus. This gender gap in vaccine sentiment highlights that females and males often conceptualise health interventions differently based on varied information sources, social roles, and lived experiences. However, our finding that more females were vaccinated than males in Malawi seems counterintuitive to their higher negative attitude scores. This paradox flags the complexity of translating vaccine perceptions into behaviour within gender power dynamics. Despite some hesitancy, Malawian women appear to have viewed the risks of COVID-19 as outweighing their concerns about the vaccine's safety. Their uptake exceeded men, likely because women shoulder greater responsibilities as caregivers and thus prioritise protecting their families and communities^26^. This aligns with evidence that African women demonstrate better health-seeking behaviour despite facing constraints in access to care and health literacy^31^.

Malawi has faced significant challenges in achieving high COVID-19 vaccine coverage compared to targets. Official statistics indicated only 0.2–14% of the population was vaccinated with 1–3 doses as of mid-2022^32^. Our study found a self-reported full vaccination rate of around 12–20% (1–2 doses) among adults, which is higher than national estimates. This discrepancy is likely because our sample was predominantly urban and semi-urban areas with greater vaccine access than rural communities. For example, a similar study by Aron et al.^23^ in Blantyre, Malawi's second-largest city, found that 19% of adults were completely vaccinated. Malawi's urban–rural divide and age-specific sociocultural barriers impede universal COVID-19 vaccine coverage. Worldwide, remote rural communities with limited health infrastructure have lower vaccination rates than some major cities^33^.

Our study's higher COVID-19 vaccine uptake among older adults than younger adults mirrors national trends in Malawi. Our finding that adults above 50 were significantly more likely to be vaccinated than those aged 18–35 years aligns with evidence that older people have appropriately prioritised getting vaccinated due to their higher risks of severe disease and mortality from COVID-19^9^. On the contrary, younger citizens, especially women of reproductive age, have demonstrated greater hesitancy and lower vaccine demand. This appears heavily driven by widespread fears that the COVID-19 vaccines could negatively impact fertility, menstruation, and future pregnancy outcomes. Such rumours and misinformation spread predominantly through social media have disproportionately affected vaccine acceptance among younger women in Malawi and globally^16^. However, older women beyond childbearing age face minimal risks of the vaccine affecting their fertility or pregnancy, removing a significant deterrent affecting younger females. Additionally, older adults in Malawi have lived through deadly past epidemics like HIV/AIDS and cholera, possibly enhancing their risk perceptions and trust in public health interventions^24^. Therefore, while younger citizens have greater access to technology and information, this has paradoxically made them more vulnerable to influential anti-vaccine propaganda.

Interestingly, participants from the semi-urban Dowa district expressed more positive attitudes towards COVID-19 vaccines than those from the more urban Zomba city in Malawi. As a semi-urban district, Dowa has stronger community and traditional leadership structures, which may have allowed positive messaging about vaccines to spread effectively via trusted local influencers. Zomba's greater individuality may have made residents more isolated and susceptible to misinformation. Further, Dowa has an older population that is more at risk from COVID-19; hence, they are more motivated to get vaccinated. Zomba has more youth who are more hesitant globally^24^. Paradoxically, higher education levels in Zomba may also expose residents more to conspiracy theories and anti-vaccine rhetoric on social media.

Community and peers are also factors that influence people’s attitudes towards vaccines. Although a significantly high number of the participants had a positive attitude towards the vaccine, other participants demonstrated that their negative attitudes towards the vaccine were born out of fear of reaction from peers and the community. Similarly, people with positive attitudes towards the vaccine expressed that peers and the community had influenced them. The participants displayed a herd mentality born from the need to preserve their right to belong in the community. For instance, many participants disagreed when asked if they would disclose to their community if COVID-19 infected a family member. This might partly be because of Malawian culture's collectivist nature and the fear of stigma and discrimination. According to Kreps et al.^34^, people naturally need belonging and are more likely to accept beliefs and convictions that align with the community. People tend to believe sensationalism, rumours, and anecdotes from social interactions^35^. Singh and Misra^35^ note that the relentless amount of information available to the masses has led to herd mentality and damaging herd behaviour.

## Conclusion and study limitations

This study examined COVID-19 vaccine knowledge, attitudes, and uptake among 394 adults across three districts in Malawi. Encouragingly, knowledge and attitudes related to vaccines were moderately positive overall. However, only around one-fifth of participants were fully vaccinated, highlighting significant vaccine hesitancy and barriers. Apparent disparities emerged across groups. Women harboured more critical concerns than men, fearing risks to fertility and pregnancy despite getting vaccinated more. Youth were more susceptible to rumours and disinformation. High levels of knowledge and positive attitudes strongly predicted vaccination likelihood but were uneven. Mobilising youth advocates and combating dangerous rumours on social media will counter distrust among younger citizens. Tailored risk communication, community participation in vaccine delivery, strengthening healthcare systems, and rebuilding public trust will be instrumental to converting vaccine acceptance into equitable nationwide coverage essential for mitigating COVID-19. Further qualitative and longitudinal research should explore nuances behind vaccine knowledge, attitudes, and behaviours over time and across regions. Variations across gender, age, geography, and socioeconomics underscore the value of contextualised investigations. The COVID-19 pandemic has highlighted vaccines as fundamental public health tools globally, but their benefits are only fully harnessed when uptake is high across all population groups. Malawi’s goal of 70% coverage is attainable if evidence-based strategies foster community confidence and demand.

Several study limitations warrant mentioning. Firstly, given that this study was cross-sectional, we adjusted for variables available in the dataset. However, it is essential to acknowledge the potential influence of unmeasured confounding factors on our conclusions. As a result, the findings may not necessarily indicate a causal relationship. Some variables, such as information on previous COVID-19 infection, related deaths among family members, and access to health institutions (both private and public), were not included in the study but would have been essential for explaining vaccination uptake. Moreover, during the survey period, the Ministry of Health established a few vaccination sites instead of individuals accessing vaccines through regular health facilities. Additionally, the survey included only one district from each region, limiting the diversity of perspectives represented. A broader selection of districts could have enhanced the generalizability of the study findings. Furthermore, the study utilised a non-probabilistic sampling technique, which introduces the possibility of selection bias and reduces the precision of the obtained estimates. Therefore, conducting further research using a random sampling technique is recommended to mitigate this limitation and ensure more accurate and representative results. Since individuals had already been vaccinated at the time of the survey, there is a possibility that they already possessed a positive attitude toward the vaccine. Nonetheless, we assume that in the past, they initially held a positive attitude toward the vaccine, motivating their decision to get vaccinated. This assumption may suggest that their attitude may have preceded the decision to get vaccinated. While our study primarily focused on estimating proportions for our outcomes of interest, we acknowledge that the sample size calculation did not directly account for the analysis of factors associated with outcomes. Future research may benefit from recalculating the sample size based on detecting minimum effect sizes for identifying associated factors. Finally, while sample size allocation was proportional to district populations, other factors at the community level, such as socio-economic status and outcome prevalence, may still influence the precision of district-specific estimates.

### Supplementary Information

Supplementary Table 1.

## Data Availability

The datasets analysed during the current study are available from the corresponding author upon reasonable request.
