# Supplementary material for: Knowledge, attitudes and demographic drivers for COVID-19 vaccine hesitancy in Malawi
Source: Sci Rep. 2024 Apr 26;14:9578. doi: 10.1038/s41598-024-60042-5 (PMC11053073; doi:10.1038/s41598-024-60042-5)
Supplement: Supplementary file 1 — Supplementary Table 1. [file 41598_2024_60042_MOESM1_ESM.docx]

**Data Collection Tools- Attitudes towards Vaccines in Malawi**


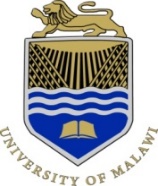


**KNOWLEDGE AND ATTITUDES TOWARDS COVID-19 AND ITS VACCINE IN MALAWI**

**Informed Consent Form**

**Purpose of the Study:** This study aims to assess knowledge and attitudes towards the Covid-19 pandemic and its vaccine in Malawi.

**What will be done?** You will be interviewed or participate in a group discussion. The interview or group discussion will take about thirty (30) minutes. During the interview or group discussion, you will be asked about your knowledge and attitudes towards the Covid-19 pandemic and its vaccine. We may ask to record some of the interviews.

**Benefits of this Study:** You will contribute to the science and knowledge about the Covid-19 pandemic and its vaccine in Malawi, and the information you will provide will inform the different interventions that other organisations and we will undertake based on the results of this study. You will also exercise your autonomy and take an active role in bringing an understanding of attitudes towards the Covid-19 pandemic and its vaccine in Malawi.

**Risks or discomforts:** No risks or discomforts are anticipated from taking part in this study. But if you feel uncomfortable with a question, you can skip that question or withdraw from the study altogether. If you decide to quit before you have finished the interview, your answers will NOT be recorded.

**Confidentiality:** Your responses will be kept entirely confidential. Each participant will be assigned a participation number, and only the participant number will appear with your responses. Only the researchers will see your responses.

**The decision to quit at any time:** Your participation is voluntary; you are free to withdraw your participation from this study at any time. You may also choose to skip any questions that you do not wish to answer.

**How the findings will be used:** The study's results will inform Covid-19 awareness campaigns in Malawi. In addition, the results of this study may be presented in educational settings and at professional conferences, and the results might be published in a professional journal.

**Contact information:** If you have concerns or questions about this study, please get in touch with the Principal Investigator, Dr Yamikani Ndasauka, +265 997 467 877 or the Chairperson of UNIMA Research Ethics Committee, Prof. Alister Munthali, +265 888 822 004

I……………………………………………………. acknowledge that I have read this information and agree to participate in this research on (date) ……………………………………………

Signature/Fingerprint of the Participant ……………………………


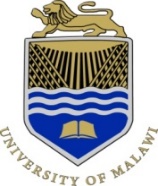


**MY VACCINE, OUR PROTECTION PROJECT**

**KNOWLEDGE AND ATTITUDES TOWARDS COVID-19 AND ITS VACCINE IN MALAWI**

**Survey Questionnaire**

1. **IDENTIFYING INFORMATION**

| 1. Name of Enumerator |  |
| --- | --- |
| 2. Date of interview |  |
| 3. District |  |
| 4. Village |  |

1. **GENERAL RESPONDENTS INFORMATION**

| **Variable** | **Response** | **Codes** |
| --- | --- | --- |
| Demographic data | | |
| 1. Gender |  | 1=Male; 2=Female; 3=Other |
| 1. Age |  |  |
| 1. Highest level of education |  | 1=no formal education, 2=primary education, 3=secondary education, 4=tertiary education |
| 1. Religion |  | 1=Christian, 2=Muslim, 3= Other  If Other (specify)_________________________ |
| 1. Marital status |  | 1=Never married, 2=Married, 3=Widowed, 4=Separated/Divorced |
| 1. Occupation |  | 1=Unemployed; 2=Employed; 3= self-employed |

1. **KNOWLEDGE AND ATTITUDES TOWARDS THE COVID-19 VACCINE**

| **Questions** | **Response** | **Codes** |
| --- | --- | --- |
| 1. Have you heard about the COVID-19 vaccine? |  | 0=No; 1=Yes ……… if No skip to section D |
| 1. What does the Covid-19 vaccine prevent? |  | 1= It prevents one from catching Covid-19;  2= It prevents one from getting severely sick from Covid-19;  3=It does not prevent one from anything  4= It prevents one from being fertile |
| 1. Even though there is a vaccine, other preventive measures are important. |  | 1= strongly agree 2= agree, 3= Neutral, 4= disagree, 5=strongly disagree |
| 1. The COVID-19 vaccine is safe. |  | 1= strongly agree 2= agree, 3= Neutral, 4= disagree, 5=strongly disagree |
| 1. The COVID-19 vaccine is essential to me |  | 1= strongly agree 2= agree, 3= Neutral, 4= disagree, 5=strongly disagree |
| 1. The COVID-19 vaccine is essential to my community |  | 1= strongly agree 2= agree, 3= Neutral, 4= disagree, 5=strongly disagree |
| 1. Every Malawian must be vaccinated. |  | 1= strongly agree 2= agree, 3= Neutral, 4= disagree, 5=strongly disagree |
| 1. Would you recommend that your families and friends get the COVID-19 vaccine? |  | 0=No, 1=Yes, 2=Maybe |
| 1. I will prefer to acquire immunity against COVID-19 naturally |  | 1= strongly agree 2= agree, 3= Neutral, 4= disagree, 5=strongly disagree |
| 1. I believe that in the long run, the Covid-19 vaccine will affect one's health. |  | 1= strongly agree 2= agree, 3= Neutral, 4= disagree, 5=strongly disagree |

1. **UPTAKE OF VACCINATION**

| **Questions** | **Response** | **Codes** |
| --- | --- | --- |
| 1. I have received the vaccination. |  | 0=No, 1=Yes |
| 1. Which of the following vaccines did you receive? |  | 1=The one dose only  2=The one with two doses |
| 1. If you got the vaccines for two doses, did you get the second dose? |  | 0=No, 1=Yes |
